# Fatty Acid and Lipopolysaccharide Effect on Beta Cells Proteostasis and its Impact on Insulin Secretion

**DOI:** 10.3390/cells8080884

**Published:** 2019-08-13

**Authors:** Paloma Acosta-Montaño, Eustolia Rodríguez-Velázquez, Esmeralda Ibarra-López, Héctor Frayde-Gómez, Jaime Mas-Oliva, Blanca Delgado-Coello, Ignacio A. Rivero, Manuel Alatorre-Meda, Jorge Aguilera, Lizbeth Guevara-Olaya, Victor García-González

**Affiliations:** 1 Departamento de Bioquímica, Facultad de Medicina Mexicali, Universidad Autónoma de Baja California, 21000 Mexicali, Mexico; 2 Facultad de Odontología, Universidad Autónoma de Baja California, 22390 Tijuana, Mexico; 3 Tecnológico Nacional de México/I.T. Tijuana, Centro de Graduados e Investigación en Química-Grupo de Biomateriales y Nanomedicina, 22510 Tijuana, Mexico; 4 Hospital General de Zona No. 30, Instituto Mexicano del Seguro Social, 21100 Mexicali, Mexico; 5 Instituto de Fisiología Celular, Universidad Nacional Autónoma de México, 04510 Ciudad de México, Mexico; 6 Tecnológico Nacional de México/I.T. Tijuana, Centro de Graduados e Investigación en Química, 22000 Tijuana, Mexico; 7 Cátedras CONACyT- Tecnológico Nacional de México/I.T. Tijuana. Centro de Graduados e Investigación en Química-Grupo de Biomateriales y Nanomedicina, 22000 Tijuana, Mexico

**Keywords:** fatty acids, lipopolysaccharides, β-cells, proteostasis, insulin secretion

## Abstract

Metabolic overload by saturated fatty acids (SFA), which comprises β-cell function, and impaired glucose-stimulated insulin secretion are frequently observed in patients suffering from obesity and type 2 diabetes mellitus. The increase of intracellular Ca^2+^ triggers insulin granule release, therefore several mechanisms regulate Ca^2+^ efflux within the β-cells, among others, the plasma membrane Ca^2+^-ATPase (PMCA). In this work, we describe that lipotoxicity mediated mainly by the saturated palmitic acid (PA) (16C) is associated with loss of protein homeostasis (proteostasis) and potentially cell viability, a phenomenon that was induced to a lesser extent by stearic (18C), myristic (14C) and lauric (12C) acids. PA was localized on endoplasmic reticulum, activating arms of the unfolded protein response (UPR), as also promoted by lipopolysaccharides (LPS)-endotoxins. In particular, our findings demonstrate an alteration in PMCA1/4 expression caused by PA and LPS which trigger the UPR, affecting not only insulin release and contributing to β-cell mass reduction, but also increasing reactive nitrogen species. Nonetheless, stearic acid (SA) did not show these effects. Remarkably, the proteolytic degradation of PMCA1/4 prompted by PA and LPS was avoided by the action of monounsaturated fatty acids such as oleic and palmitoleic acid. Oleic acid recovered cell viability after treatment with PA/LPS and, more interestingly, relieved endoplasmic reticulum (ER) stress. While palmitoleic acid improved the insulin release, this fatty acid seems to have more relevant effects upon the expression of regulatory pumps of intracellular Ca^2+^. Therefore, chain length and unsaturation of fatty acids are determinant cues in proteostasis of β-cells and, consequently, on the regulation of calcium and insulin secretion.

## 1. Introduction

Type 2 diabetes mellitus (T2DM) is a metabolic disorder characterized by dysfunction and progressive loss of pancreatic β-cells, which are responsible for insulin synthesis, originating tissue damage and several long-term complications [1]. This pathology could occur in conditions of obesity accompanied with a decrease in cellular response of insulin-dependent tissues. Phenomena such as β-cells proliferation and hyperinsulinemia seem to have a compensatory effect. However, these conditions possibly lead to β-cell dysfunction [2]. Indeed, in obesity and insulin resistance, β-cells are able to modulate the energetic metabolism in order to increase insulin synthesis and release [3].

Packaged in small granules, each β-cell has approximately 10,000 granules of insulin, of which only a fraction is released in response to an increase in glycemia [4,5,6]. After translation, pre-proinsulin polypeptides are translocated to the endoplasmic reticulum (ER) to acquire the native folding [7]. However, under conditions of high protein demand, ER stress is triggered. Therefore, the transducers of the unfolded protein response (UPR), IRE1, ATF6α, and PERK, promote the transcription and translation of target chaperones through XBPIs, ATF6α and CHOP transcription factors, respectively, when unfolded proteins accumulate in the lumen [8,9]. The three branches of UPR are essential in β-cells homeostasis to reduce ER stress and to ensure adequate synthesis of peptides such as insulin [6].

The key triggering factor for release of insulin granules is the increase of intracellular Ca^2+^, associated with increased extracellular glucose [10]. Several mechanisms regulate the flux of Ca^2+^ in cells, such as the plasma membrane Ca^2+^-ATPase (PMCA) and the Na^+^/Ca^2+^ exchanger (NCX) [11]. PMCA belongs to the P-type ATPases whose function is critical to maintain the concentration of cytosolic calcium below 300 nM [12]; it is encoded by four different genes (*ATP2B1*-*ATP2B4*) and the isoforms expressed differentially in excitable and non-excitable cells [13,14]. On the other hand, the NCX uses the electrochemical Na^+^ gradient to export Ca^2+^ against gradient concentration [15]. Four isoforms have been identified for NCX (NCX1-4) and NCX1 is the most expressed in rat β-cells. The role of PMCA and NCX1 in β-cells is critical; both contribute to the extrusion of Ca^2+^, therefore to the regulation of insulin release.

Lipotoxicity is associated with deleterious effects due to an increase of circulating fatty acids when the storage capacity of triglycerides in adipose tissue is overloaded. Indeed, lipotoxicity is related to β-cell dysfunction, wherein the capacity of protein folding may be compromised [16]. Lipotoxicity represses the protein trafficking from ER to the Golgi apparatus [17,18]. The palmitic (PA) and oleic (OA) fatty acids have been reported to induce apoptosis of β-cells, but only palmitic acid activates the PERK and IRE1 pathways [19]. Although the mechanism responsible for lipoapoptosis induced by ER stress is complex, it could involve alterations in Ca^2+^ [20]. Dysfunction of pancreatic β-cells has been associated with a low-grade inflammatory condition (meta-inflammation) associated with the excess of white adipose tissue in obesity [21]. It has been proposed that lipopolysaccharides (LPS) activate a signaling pathway mediated by TLR-4 [22], and this has been identified as a factor in obesity-associated metabolic disorders [23]. Additional evidence suggests that patients with T2DM show an altered intestinal microbiota in which Gram-negative bacteria predominate, then LPS, a critical component of its outer membrane, might promote inflammation [24]. Accordingly, the gut microbiota may play a role as a risk factor in the development of T2DM [25].

In this work, the effect of LPS on the UPR pathway in addition to free fatty acids was characterized, which modifies several key processes related to insulin secretion. Specifically, the properties that provide to fatty acids with their ER stress-inducing or protective role with a concomitant effect of LPS were described, through modulation of specific targets that regulate homeostasis of proteins (proteostasis), and then insulin secretion.

## 2. Materials and Methods

### 2.1. Reagents

RPMI-1640 medium (Gibco, Waltham, MA, USA), fetal bovine serum (FBS), penicillin, streptomycin, amphotericin B, palmitic acid (PA), oleic acid (OA), palmitoleic acid (PAO), stearic acid (SA), myristic acid (MA), lauric acid (LA), fatty acid-free bovine serum albumin (BSA) (Fraction V), lipopolysaccharides (from *Escherichia coli* O111:B4), trypsin, phosphate buffer saline (PBS), (3-(4,5-Dimethylthiazol-2-yl)-2,5-Diphenyltetrazolium Bromide (MTT) and tunicamycin (Tum) were obtained from Sigma-Aldrich.

### 2.2. Cell Culture

The established rat β-cell line RIN-m5F (American Type Culture Collection, ATCC, Manassas, VA, USA) was grown in RPMI-1640 medium supplemented with 10% FBS, 10 U/mL penicillin, 10 µg/mL streptomycin and 25 µg/mL amphotericin B. Cultures were maintained at 37 °C in a humidified atmosphere of 95% air and 5% CO_2_. The culture medium was changed every 3 to 4 days and also passaged once per week, according to ATCC recommendations.

### 2.3. Fatty Acid and LPS Preparation

Stock solutions were prepared as follows: fatty acids were dissolved in ethanol:H_2_O (1:1, vol:vol) at 60 °C at a final concentration of 75 mM. Stock solutions were complexed with fatty-acid-free BSA (3 mM in H_2_O) by stirring for 1.5 h at 37 °C, then diluted in culture media and filtered by a 0.22-µm pore membrane. The final molar ratio of fatty acid:BSA was 4:1 with a final concentration of 300 µM BSA. LPS O111:B4 were dissolved in ultrapure water (1 mg/mL), and then diluted to a final concentration of 100 ng/mL, as previously reported [26].

### 2.4. Cell Viability Assays

Cell viability was evaluated using the MTT assay according to previous protocols [27]. Formazan crystals were dissolved in a lysis buffer containing 20% SDS and 50% N,N-dimethylformamide (pH 3.7) for 12 h at 37 °C, and absorbance readings were acquired at 560/530 nm.

### 2.5. Western Blotting Analysis

Proteins were extracted from cell cultures under different conditions, using ice-cold protein lysis buffer (150 mM NaCl, 10 mM Tris, pH 7.4, 1% Triton X-100, 0.5% NP40, 1 mM EDTA, 1 mM EGTA, 0.2 mM sodium orthovanadate, 10 mM benzamidine, 10 µg/mL leupeptin, 10 µg/mL aprotinin, and 250 µM PMSF). An average of 25 µg of protein samples from RIN-m5F cells were separated on 10% SDS-PAGE electrophoresis and transferred to polyvinylidene difluoride (PVDF) membranes. The membranes were blocked with 5% non-fat milk in Tris-buffered saline 0.1% Tween-20 (TBS-T) for 1 h at 37 °C, and incubated at 4 °C overnight with primary antibody (anti-XBP1s, anti-c-Jun, anti-ATF6α, anti-CHOP, anti-PMCA1/4, anti-Lamin-B, anti-β-adaptin, anti-BiP, anti-SPT, anti-SERCA2, anti-PMCA3, anti-NCX1, and anti-β-actin). Following washing with TBS-T, the membranes were further incubated for 1 h at 37 °C with the corresponding horseradish peroxidase-conjugated secondary antibodies. Proteins were detected with the enhanced chemiluminescence reagent (Immobilon Western from Millipore, Burlington, MA, USA).

### 2.6. Immunoprecipitations on Nucleus Extracts

Nuclei separation was carried out using a buffer containing sucrose (250 mM) and imidazole (3 mM) pH 7.4, supplemented with protease and phosphatase inhibitors. Cells were scraped from culture dishes and 21 passages were performed through a 22-G syringe. For the recovery of nuclei, lysates were centrifuged at 3400 rpm for 15 min. Then, nucleus fractions (200 μg) were incubated with an anti-c-Jun antibody (1:400) for 2 h at 4 °C. Immune complexes were precipitated with protein G agarose Fast Flow (Millipore) 12 h at 4 °C. Immuno-precipitated proteins were washed 3 times and suspended in Laemmli buffer, separated by SDS-PAGE gels and transferred to PVDF membranes for Western-blot analysis. Protein detection was performed based on a previous protocol [26].

### 2.7. Insulin ELISA Assays

Cells were proliferated in 20 mm cell culture plates at a density of 2.3 × 10^5^ cells/mL (2 mL). Cells were maintained in proliferation for 72 h, and later different treatments were performed on a volume of 1 mL. Cell culture medium was recovered, and centrifuged for 5 min at 5000 rpm. Supernatant medium was recovered and diluted (1/3) in PBS. Insulin concentrations were quantified with the Rat Ultrasensitive Insulin ELISA kit (80-INSRTU-E01, E10; ALPCO Diagnostics, Salem, NH, USA) through several adaptations according to manufacturer recommendations. Absorbance readings were performed at 450 nm, and results were reported as ng/mL.

### 2.8. Immunocytochemistry Assays

Treated cells were fixed in ethanol:acetone (1:1) at −20 °C and permeabilized with 0.01% Triton X-100. Slides were processed for immunocytochemical detection of transcription factors XBP1s and CHOP using the mouse/rabbit Polydetector HRP/DAB (BioSB, Santa Barbara, CA, USA). Slides were blocked with a horseradish peroxidase-conjugated antibody for 5 min and 1% bovine serum for 20 min, then incubated at 4 °C with the primary antibody. Following washing with PBS, the slides were further incubated for 40 min at room temperature with the secondary antibody. Immunostaining was performed with free-floating diaminobenzidine (DAB) for 2 min. After stopping the reaction, sections were embedded in Entellan and analyzed with the Axiovert.A1 FL-LED Microscope (Carl Zeiss, Oberkochen, Germany) and AxioCam ERc 5s (Carl Zeiss, Oberkochen, Germany). Image acquisition software ZEN-Blue edition was employed for image processing (Carl Zeiss, Oberkochen, Germany).

### 2.9. Fractionation of Cultured Cells and Determination of Triglycerides

Cells were processed as elsewhere reported [28]. Briefly, cells were washed with PBS 1×, scraped from the plate and transferred into a 2-mL tube, then centrifuged at 2000× *g* for 2 min. The pellet was resuspended in 200 µL of 60% sucrose dissolved in lysis buffer (10 mM HEPES and 1 mM EDTA, pH 7.4). After mixing, samples were incubated on ice for 10 min. Next, 800 µL of ice-cold lysis buffer was added, and incubated on ice for another 10 min. Cells were lysed by 5 passes through a 27-G needle and centrifuged at 100× *g* for 2 min. A mixture consisting of 2 µL of methylene blue mix per mL of lysis buffer was prepared. Then, 600 µL of this mixture were carefully layered on top of the cell homogenate and centrifuged at 20,000× *g* at 4 °C for 2 h. Tubes were frozen at −70 °C, the dye layer containing lipids (floating layer) was recovered by cutting the tube and transferred to a new tube where 100 µL of lysis buffer was added. Triglyceride content of fractions was measured using the Triglyceride-LQ kit (Giorna, Spain). 

### 2.10. Calcium Quantification

Cells were processed according to a previous protocol [29] and intracellular calcium levels were calculated according to Patel et al. [30]. After treatments, cells were washed with PBS 1× and incubated at 37 °C with Opti-MEM/1.5 µM Fura-2/AM (fluorescent Ca^2+^ indicator) for 75 min. Subsequently, cells were washed with PBS 1× and incubated for 20 min at room temperature. Cells were washed with PBS 1×, and fluorescence measurements were carried out at 340 and 380 nm excitation wavelengths, and a 510 nm emission wavelength, employing a Cary Eclipse Fluorescence Spectrophotometer (Santa Clara, CA, USA).

### 2.11. Nitrite Assay

Nitrites were measured in the extracellular culture medium from treated cells as an index of reactive nitrogen species (RNS) formation, using the Griess reaction (40 mg/mL). Absorbance readings were performed at 450 nm in an ELISA plate.

### 2.12. Fluorescence-Probe Synthesis

Based in a methodology developed by our group, BODIPY (BDP) coupled to palmitic acid (BDP-PA), BODIPY coupled to stearic acid (BDP-SA), and BODIPY coupled to myristic acid (BDP-MA) were synthesized. Briefly, BODIPY-8-Ethylendiamine (0.436 mM) [31] in dichloromethane anhydrous (8 mL) was added to *N*’-ethylcarbodiimide hydrochloride (0.524 mM), hydroxybenzotriazole (0.524 mm), *N*-methylmorpholine (1.308 mM) and the corresponding fatty acid (0.48 mM). The mixture reaction was stirred for 5 h under argon atmosphere at 25 °C. Solvent was eliminated under reduced pressure, and the product was dissolved in water (20 mL) and extracted with dichloromethane. The combined organic extracts were washed with brine and dried over anhydrous Na_2_SO_4_, and later the product was concentrated. The crude product was purified by flash chromatography. All structures were confirmed by mass spectroscopy and nuclear magnetic resonance (NMR).

### 2.13. Confocal Microscopy

A LEICA TCS-SP8 confocal scanning biological microscope (LEICA Microsystems Heidelberg GmbH, Nussloch, Germany) was employed in order to characterize the BDP-MA, BDP-PA and BDP-SA sub-cellular localization. After different experiments with BDP-fatty acids (10–100 µM), cells were washed with PBS 1× 3 times, and then incubated with Aza-2-DBP for 30 min (0.8 µM). Later, cells were washed extensively and fixed with cold paraformaldehyde, and mounted for observation. Macroscopically different zones were recorded, preferentially at the center of the specimens, in order to depict representative images. Images were recorded at excitation/emission wavelengths of 488/495-545 and 638/650–750 nm for detection of BDP-fatty acids (green) and AZA-2-BDP (red), respectively.

### 2.14. Colocalization of ER-Tracker Probe and BDP-SFA

RIN-m5F cells were proliferated to 90% of confluence, and treated with BDP-PA (5–25 μM) for 12 h. Later, culture cells were washed with HBSS buffer and then ER-tracker (1 µM) was added and incubated for 25 min at 37 °C. Cells were washed once with HBSS buffer and fixed with 4% formaldehyde, for 2 min at 37 °C. Finally, cells were washed twice with PBS 1×. Images were recorded at excitation/emission wavelengths of 488/495-545 and 587/615 nm for detection of BDP-PA (green) and ER-tracker (red), respectively.

### 2.15. Quantitative PCR Methods

Total RNA from RIN-5mF cells was obtained with Trizol following the supplier’s instructions. cDNA was synthesized using 1 µg of RNA and the iScript RT Supermix from Bio-Rad (Hercules, CA, USA). cDNA concentration was standardized to be used for qPCR with the PowerUp Sybr Green Master Mix 2X (Applied Biosystems) according to manufacturer’s instructions. Primer sequences were: *RPL13*fwd 5′-AAAGCCTGCCAGAATTTCTCT-3′, *RPL13*rev 5′-GGGCTCCAAGGACTCTCTG-3′, *ATP2B1*fwd: 5′-GCAATTGAAAATCGCAACAA-3′; *ATP2B1*rev: 5′-TTCAGAGGCTGCATCTCCAT-3′; *ATP2B2*fwd: 5′-CGGAGTGTGGACTAAC AGCA-3′, *ATP2B2*rev: 5′-CTAGGGACTGCGTGACCAAG-3′; *ATP2B3*fwd: 5′-CAATCTTTCTA CCCCTACTCACATC; *ATP2B3*rev: 5′-GATGCAACTCTTGGATTCTGG; *ATP2B4*fwd: 5′-CTCTG AAAATCGCAACAAAGC-3′, *ATP2B4*rev: 5′-AGTGGCTGGATTTCCAAGG-3′. qPCR reactions were performed in triplicate using an ABI PRISM 7000. qPCR data were analyzed with the 2^−ΔΔCt^ method with *RPL13* (ribosomal protein L13) as reference calibrator, and reported as fold change.

### 2.16. Statistical Analysis

Data are expressed as mean ± SD. The statistical analyses were conducted with one-way ANOVA, and differences among means were compared with the Bonferroni test using a level of significance of *p* < 0.001 unless otherwise specified. The software used was GraphPad Prism version 6 (San Diego, CA, USA). For the analysis of the expression of proteins by Western blot, a semiquantitative analysis was performed using β-actin as loading control with the software ImageJ (Bethesda, MD, USA). Likewise, ImageJ was employed in the processing of immunocytochemistry results.

## 3. Results

**Figure 1:**
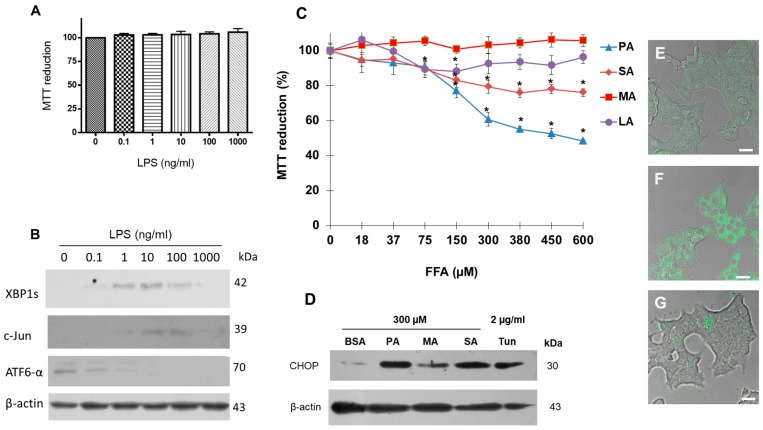
Lipopolysaccharides (LPS) and saturated fatty acids (SFA) trigger the unfolded protein response (UPR) pathway in β-cells, with 16C-pamitic acid (PA) the most lipotoxic. (**A**) Effect of increasing concentrations of LPS (0.1–1000 ng/mL) on cell viability. (**B**) Under the same conditions, expression of proteins XBP1s, c-Jun, and ATF6α analyzed by Western blot. (**C**) Effect of the treatment of saturated PA, stearic acid (SA), myristic acid (MA) and lauric acid (LA) (0–600 µM) on cell viability. (**D**) Effect of SFA treatment on CHOP expression. Tunicamycin (Tum) was used as a control. (**E**) Cellular distribution of BODIPY (BDP)-MA probe (green channel) (**E**), BDP-PA (**F**) and BDP-SA (**G**) in β-cells. Bars correspond to 20 μm. In panel B and D, β-actin was used as a loading control. In panel A and C, mean values are presented (*n* = 6, mean ± SD), * *p* < 0.0005 with respect to control.

First, we examined the effects of LPS upon the UPR pathway in β-cells. Although increasing concentrations of LPS did not modify cell viability (Figure 1A), LPS promoted an increase in the expression levels of transcription factors associated with diminishing ER stress, such as XBP1s and c-Jun. However, the ATF6α branch that showed a basal expression in the control condition showed a diminished expression by effect of LPS treatment (Figure 1B). Upon proving that LPS cause ER stress through the modification of the UPR pathway without affecting cell viability, we decided to evaluate the effect of the most representative saturated fatty acids (SFA). The four main saturated fatty acids (SFA) found in the phospholipid membranes and neutral lipids—lauric acid, myristic acid, palmitic acid, and stearic acid—were studied in order to characterize the role of the fatty acid acyl-chain length on β-cell physiology. In a first approach, we evaluated the effect of increasing concentrations of SFA (0–600 µM) on cell viability, considering a ratio of albumin/SFA of 1/4, which is in the range of reported plasmatic concentrations of fatty acids (350–500 μM) [32]. Results suggest PA as the most lipotoxic fatty acid, followed by SA, as demonstrated by the cellular viability measured by the MTT assay (Figure 1C), which is associated with mitochondrial activity. Although the UPR pathway was activated by the three SFA tested (Figure 1D), palmitic acid showed the major effect on CHOP expression. In this case, tunicamycin (Tun) was used as a positive control inducing ER stress, by inhibition of N-glycosylation protein process [33].

In order to evaluate the intracellular distribution of SFA, our working group carried out the chemical synthesis of three fluorescent probes to be tracked by confocal microscopy: BODIPY-PA (referred to as BDP-PA), BODIPY-SA (referred to as BDP-SA) and BODIPY-MA (referred to as BDP-MA). After the treatments, the internalization of BDP-PA (Figure 1F) was higher compared to detection with BDP-MA or BDP-SA (Figure 1E,G). This phenomenon supports the results obtained for cell viability, wherein PA was the most lipotoxic fatty acid.

**Figure 2:**
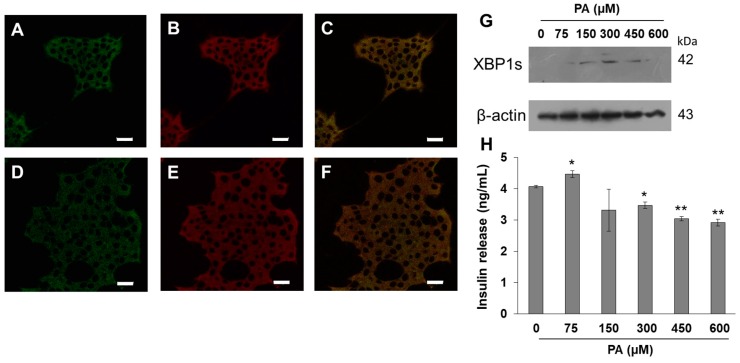
BDP-PA probe colocalizes with endoplasmic reticulum (ER)-tracker, and UPR is activated. (**A**,**D**) Confocal images of β-cells treated with BDP-PA (green channel). (**B**,**E**) Localization of ER-tracker probe (1 μM) (red channel). (**C**,**F**) Images corresponding to the merge. Bars correspond to 20 µm. (**G**) Under increasing concentrations of PA, UPR activation was evaluated through XBP1s activation, as well as the insulin release (**H**). Mean ± SD are presented (*n* = 3), * *p* < 0.05, **** *p* < 0.005.

PA is the most abundant SFA in the human body distributed in neutral lipids, the phospholipids of plasma membrane, organelles such as ER, and as a free fatty acid bound to albumin in plasma. Although the UPR activation by the three fatty acids was observed, PA treatment was more evident in the activation of UPR. In the endoplasmic reticulum, one-third of cellular proteins are synthesized, membrane and extracellular proteins are also folded, and, importantly, the ER is the main site of calcium storage [34,35]. In order to characterize the cellular distribution of PA, we employed the specific probe ER-tracker, performing the co-localization of ER-tracker (red channel) and BDP-PA (green channel) (Figure 2A–E). Results show that BDP-PA is located in the endoplasmic reticulum. In a complementary way, under a PA dose-dependent stimulus (0–600 µM), we corroborated the activation of IRE1 branch of the UPR through XBP1s expression (Figure 2G), and concomitantly the effect on insulin secretion function (Figure 2H). At 150 and 300 μM PA, the UPR activation was more evident, which agrees with the drop of cell viability observed previously.

**Figure 3:**
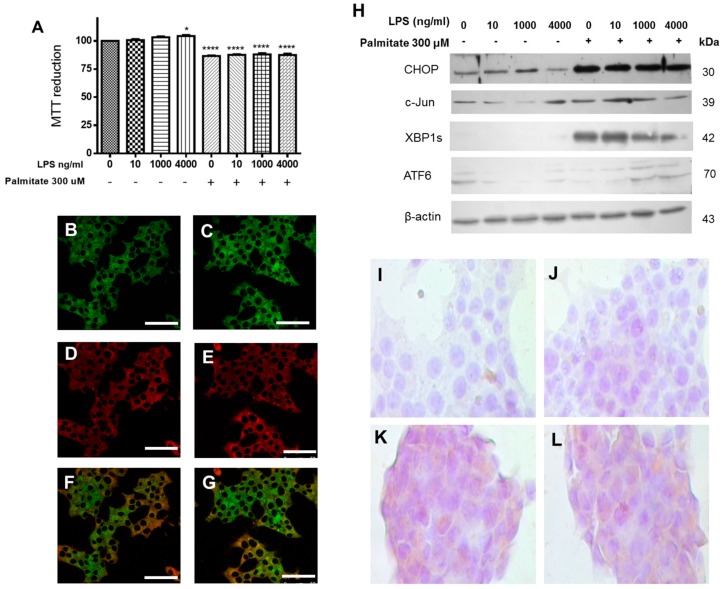
The combined treatment of LPS and PA potentiate the UPR activation in β-cells. (**A**) Effect of treatments with LPS and PA on cell viability (*n* = 6, mean ± SD), * *p* < 0.05, **** *p* < 0.001. Confocal images of β-cells treated with BDP-PA (**B**), Aza-2-BDP (**D**) and the merge (**F**). Under the same conditions, β-cells treated with BDP-PA plus LPS (**C**), Aza-2-BDP plus LPS (**E**), and the merge (**G**). Bars correspond to 50 μm. (**H**) Expression of CHOP, c-Jun, XBP1s, and ATF6α under increasing concentrations of LPS and PA (300 µM). β-actin was used as a loading control. (**I**,**J**) Detection of XBP1s by immunocytochemistry in control cells (negative signal). (**K**,**L**) Detection of XBP1s on PA-treated cells, positive signal (++) is determined by brown areas.

Aiming to elucidate the combined effect of saturated fatty acids and LPS on cell viability as well as their intracellular location, which has not been described in the literature so far, β-cells were treated with PA and LPS. Cell viability was evaluated after a 24-h incubation with 300 μM palmitic acid and 100 ng/mL LPS, and results show a significant decrease in cell viability, as compared to controls (Figure 3A). In addition, we characterized the intracellular distribution of BDP-PA probe and Aza-2-BODIPY (used as a counterstain; both were synthesized by our group). Interestingly, the counterstain probe Aza-2-BODIPY showed a cytoplasmic localization within the β-cells. By contrast, the BDP-PA signal was rather concentrated around the perinuclear area, wherein ER is localized, a phenomenon that was slightly increased under the treatment with LPS (Figure 3B–G). These results were corroborated, now employing the ER-tracker probe (Supplementary Figure S1).

Although the LPS stimulus did not cause a decrease in cell viability, it slightly activated the UPR pathway through CHOP. However, when LPS and PA treatments were evaluated together, the UPR pathway was significantly activated by this transcription factor (CHOP). In parallel, it also increases the expression of XBP1s and ATF6a, as well as c-Jun (Figure 3H), which might be a possible response associated with the decrease in cell viability. Changes observed in cellular viability and the increase in XBP1s expression induced by PA agree with the analysis by immunocytochemistry of XBP1s (Figure 3I–L), where positive signals are observed as brown areas. Together, these data show the activation of the UPR branches induced by LPS and the SFA most abundant in neutral lipids, as well as the ER localization of PA.

**Figure 4:**
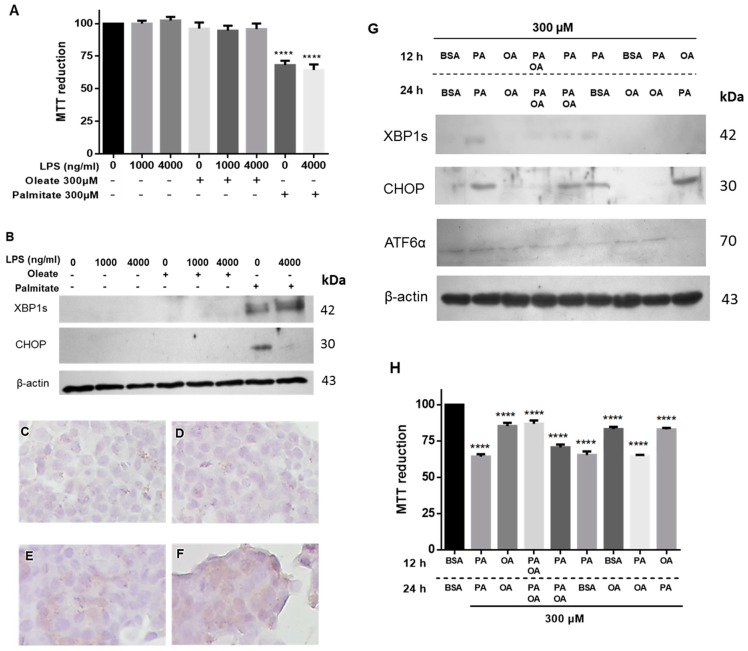
Oleic acid (OA) treatment does not trigger UPR and shows a partial protective role on lipotoxicity induced by PA. (**A**) MTT assay performed in β-cells exposed to different concentrations of LPS in the presence of 300 µM of PA or OA. (**B**) Under the same conditions, expression of XBP1s and CHOP. (**C**,**D**) Detection of CHOP by immunocytochemistry in control cells (negative signal). (**E**,**F**) Immunocytochemistry for CHOP under LPS and PA stimuli, positive signal (+) corresponds to brown areas. (**G**) Characterization of XBP1s, CHOP and ATF6α under a pre-treatment with fatty acids for 12 h, followed by the next treatment. (**H**) Under the same conditions, cell viability was characterized. β-actin was used as a loading control. In panel (**A**) and (**H**), (*n* = 6, mean ± SD), **** *p* < 0.001 with respect to control.

To assess if lipotoxicity could be extended to unsaturated fatty acids, the effect of oleic acid was evaluated. Unlike palmitic acid treatment, the exposure to OA neither modified the cell viability nor caused ER stress by activation of CHOP or XBP1s (Figure 4A,B). In this case, immunocytochemistry for CHOP in the presence of LPS and PA evidenced an increase in their expression (Figure 4C–F). Considering the stressor effect of palmitic acid and that OA did not produce ER stress, the possible protective or restorative role of OA was assessed under cytotoxicity induced by palmitic acid. With both fatty acids’ stimuli, the effect of palmitic acid is reversed and cell viability is maintained (Figure 4G,H). Additionally, 12-h treatment with palmitic acid and followed by 12 h with OA alone, CHOP is not activated, but cell viability decreases (Figure 4H). However, treatment with OA prior to palmitic acid does not affect viability, but it activates CHOP, which may be a consequence of treatment with palmitic acid. These data suggest that OA could play a partial protective role.

**Figure 5:**
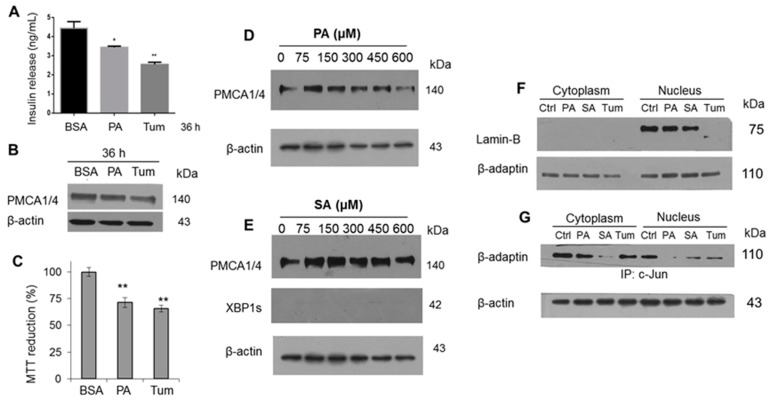
Endoplasmic reticulum stress reduces insulin secretion modifying the expression of PMCA1/4 and nuclear complexes. (**A**) Effect of treatment with PA and Tum on insulin secretion (*n* = 3, mean ± SD) * *p* < 0.05, ** *p* < 0.01. (**B**) Western blot characterization of PMCA1/4 isoforms. (**C**) Cellular viability assay, ** *p* < 0.001 with respect to control (*n* = 6, mean ± SD). Expression of PMCA1/4 and XBP1s in the presence of (**D**) PA or (**E**) SA. (**F**) Expression of Lamin-B and β-adaptin in nucleus extracts. (**G**) Evaluation of the complex formed by c-Jun and β-adaptin. β-actin was used as a loading control.

Aiming to evaluate one of the most relevant functions of β-cells, the insulin release, as we reported previously, we found a decrease in β-cell function with PA treatment, now using tunicamycin (Tum) as a control (Figure 5A). This characterization was complemented with the evaluation of CHOP, ATF6α and c-Jun in a time range (0–36 h), upon which a gradual activation was evidenced (Supplementary Figure S2). Considering that an increase of cytoplasmic [Ca^2+^] in β-cells is the triggering factor for insulin release, a slight modification in PMCA expression isoforms 1 and 4 (PMCA 1/4) was observed (Figure 5B). Contrary to what was expected, the decrease in PMCA expression may favor an increase in insulin release due to a rise in intracellular [Ca^2+^], however, other regulatory systems may be involved. The decrease in insulin secretion induced by PA matches with the cell viability decrease (Figure 5C). In order to broaden the effect of main saturated fatty acids, under a dose-dependent administration of PA, the decrease of PMCA1/4 expression was confirmed (Figure 5D), however, this change was not found in SA stimulus (Figure 5E). This phenomenon was supported through the UPR activation mediated by IRE1 only with PA treatment as previously described (Figure 2G), and not with SA treatment (Figure 5E).

The specific case of the cytotoxicity exerted by Tum may be associated with an alteration in the nuclear structure (Figure 5F), considering the involvement of Lamin-B, a similar phenomenon that we have described in macrophages treated with LPS [36]. It should be noted that our group has described the function of new protein complexes formed by the adapter protein β-adaptin and transcriptional activators such as c-Myc and c-Jun that regulate the cell cycle and mechanisms such as endocytosis [36]. This condition was found to be affected in the nucleus under the stimuli with PA and Tum (Figure 5G), which we have correlated with the presence of an oxidative imbalance [37] implicating critical functions in the cells.

**Figure 6:**
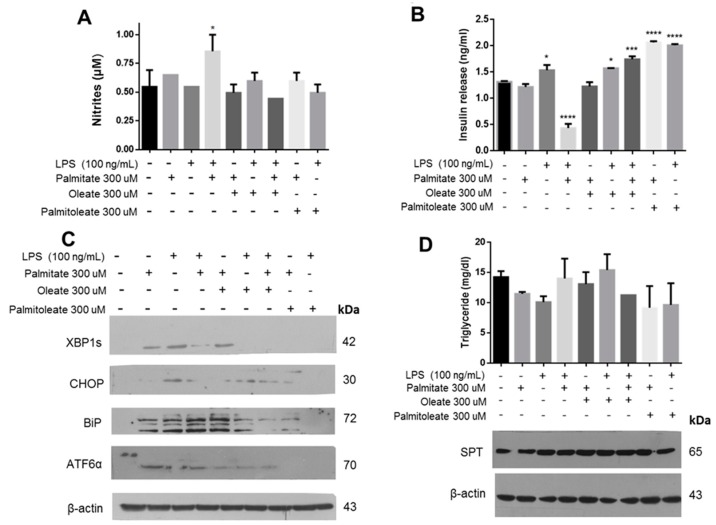
Unsaturated fatty acids could regulate the insulin secretion. (**A**) Quantification of nitrites in the extracellular medium under the incubation with several fatty acids and LPS. (**B**) Insulin secretion in cells exposed to the same treatments. (**C**) Western blot of UPR targets, XBP1s, CHOP, BiP and ATF6α. (**D**) Effect of treatments on metabolic regulation through intracellular quantification of triglycerides and evaluation of SPT. β-actin was used as a loading control. In panels (**A**) and (**B**), (*n* = 3, mean ± SD), * *p* < 0.05, *** *p* < 0.01, **** *p* < 0.001 compared to control.

Expanding the characterization of this phenomenon, we found that stimulation with PA and LPS promotes the formation of RNS in the extracellular medium (Figure 6A). In a previous publication, we correlated the formation of RNS with the oxidative damage phenomenon and UPR activation in macrophages treated with LPS [36]. In our conditions, a drastic drop in insulin release was observed upon the treatment with palmitic acid and LPS (Figure 6B), associated with an increase in RNS formation in the culture medium. In this case, the activation of BiP and XBP1s matches with an increase of nitrites (Figure 6C). Taking into account a partial protective function of OA under lipotoxicity conditions, the role of other unsaturated fatty acids such as PAO was evaluated. In both cases, the data suggest that treatment with unsaturated fatty acids prevents the drop of insulin release that is generated under the conditions of lipotoxicity by PA and LPS (Figure 6B). Interestingly, there is a mild increase of insulin release with PAO treatment with respect to controls. Data suggest that there is no metabolic effect that could modulate the treatment with unsaturated fatty acids, considering the quantification of triglycerides wherein only a slight increase in treatment of PA and LPS was registered, as well as the evaluation of targets such as serine palmitoyl-transferase (SPT), a key enzyme that uses palmitoyl-CoA and serine during ceramide synthesis (Figure 6D), which has been involved in several pathological diseases [38]. Then, our results suggest that under the cellular conditions evaluated, there was no significant metabolic alteration in β-cells.

**Figure 7:**
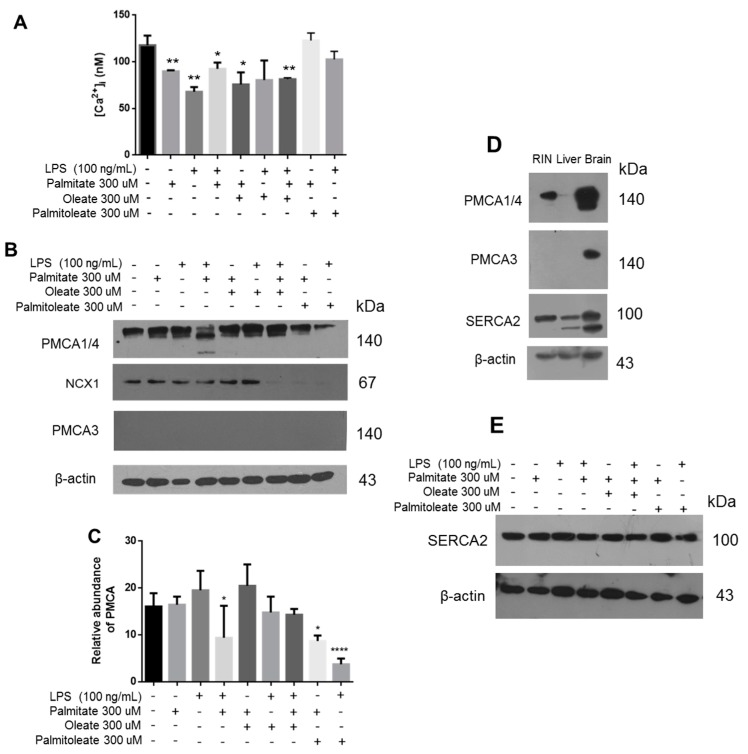
The treatment with unsaturated fatty acids regulates the expression of calcium transporting proteins. (**A**) Quantification of intracellular [Ca^2+^] under lipotoxicity conditions. * *p* < 0.05, ** *p* < 0.01 compared to control (*n* = 3, mean ± SD). (**B**) Expression of targets PMCA1/4, NCX1 and PMCA3, which are regulated by oleic and palmitoleic acid. (**C**) Quantitative characterization of the expression of PMCA1/4, the analysis was based on three different experiments. * *p* < 0.05, **** *p* < 0.001 compared to control (*n* = 3, mean ± SD). (**D**) Validation of antibodies anti-PMCA1/4, anti-PMCA3 and anti-SERCA2 in RIN-m5F cells, liver and brain tissues. (**E**) Western blot of SERCA2. β-actin was used as a loading control.

To elucidate the correlation of the lipotoxicity phenomenon directly with the main β-cell function, the intracellular [Ca^2+^] was evaluated under the same treatments (Figure 7A). We found that palmitic acid and LPS induced a decrease in [Ca^2+^], which was demonstrated to be stabilized under incubation with oleic acid. Importantly, treatment with palmitoleic acid avoids the alteration in [Ca^2+^] levels that induced the lipotoxicity. The most important proteins regulating intracellular [Ca^2+^] were assessed, which in turn could explain this phenomenon. Importantly, a decrease in the expression of isoforms 1 and 4 of PMCA was induced by the presence of palmitic acid and LPS (Figure 7B). Although these isoforms maintain a ubiquitous expression, they show a low Ca^2+^ affinity in comparison to PMCA2 and PMCA3 isoforms [39]. Specifically, a second band of lower molecular weight was detected in the Western blot of PMCA1/4 (Figure 7B). This could suggest a proteolytic cleavage mediated by calpain, which has been described to induce its degradation [40], as analyzed by our group in a previous work [41], therefore altering Ca^2+^ homeostasis, which affects key functions such as insulin secretion. Under our experimental conditions, the expression of PMCA3 in β-cells was undetectable (Figure 7B), although the expression of this isoform has been reported elsewhere [42]. The efficacy of the antibody was evaluated using brain tissue as a positive control where it is abundantly expressed, and in the liver tissue considering that the presence of transcripts has been reported before [43] (Figure 7D).

Extending our characterization associated with PMCA1/4, the treatment with OA prevented the alteration of PMCA1/4 expression, which was evidenced through the statistical analysis (Figure 7C). However, a decrease in the expression of PMCA1/4 was found under PAO treatment, without evidence of degradation (Figure 7B). Furthermore, cytoplasmic Ca^2+^ levels were maintained at the same levels with respect to controls (Figure 7A). Notwithstanding this, under these conditions an increase in insulin release was recorded (Figure 6B). In order to expand the characterization of this phenomenon, other relevant proteins regulating intracellular [Ca^2+^] were evaluated. A decrease in NCX1 expression was found under treatment with unsaturated fatty acids, which is responsible for Ca^2+^ output against the gradient (Figure 7B). This phenomenon could represent a key condition in the regulation of insulin release. With respect to the evaluation of other targets, which could contribute to the explanation of the effect with unsaturated fatty acids, importantly, significant changes were not registered in the expression of SERCA2 (Figure 7E).

**Figure 8:**
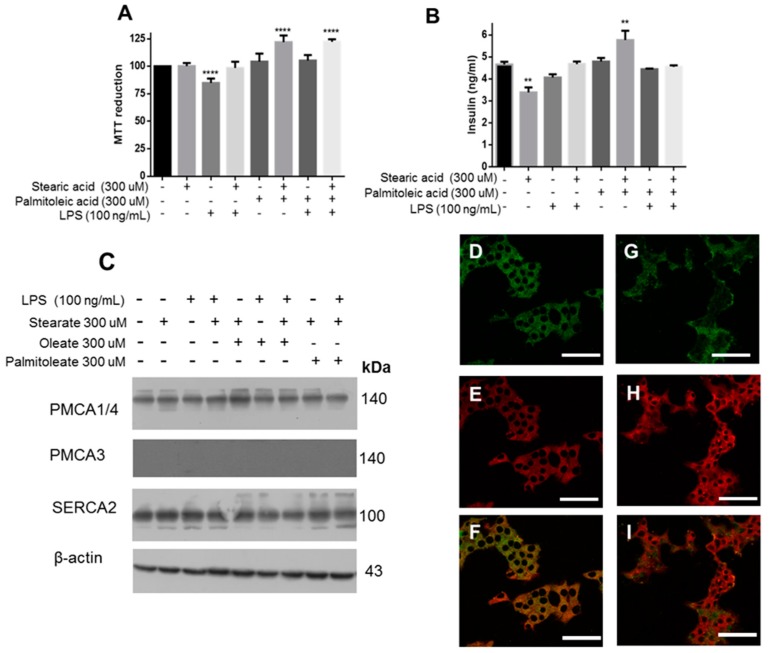
Stearic acid does not modify the homeostasis of calcium in β-cells. (**A)** Viability assay experimentation under treatment with different fatty acids. **** *p* < 0.01 with respect to control (*n* = 6, mean ± SD). (**B**) Determination of insulin concentrations in supernatant medium. ** *p* < 0.05 with respect to control (*n* = 3, mean ± SD). (**C**) Expression of targets that regulate the intracellular calcium concentration. β-actin was used as a loading control. Confocal images of β-cells treated with BDP-PA (**D**), Aza-2-BDP (**E**) and the merge (**F**). Under the same conditions, β-cells treated with BDP-SA (**G**), Aza-2-BDP (**H**), and the merge (**I**). Bars correspond to 50 μm.

Taking into account these results, the role of SA, the second most abundant SFA in biological membranes, was evaluated. In particular, the stimulation with SA did not significantly decrease cell viability, contrary to what was observed with LPS, which caused a slight decrease in the viability (Figure 8A). In fact, insulin release is only modified under SA treatment (Figure 8B), and the incubation with PAO prevents this decrease and maintains insulin levels similar to controls. Under these same experimental conditions, PMCA1/4 expression remained constant (Figure 8C) and in none of the conditions, even upon treatment with SA and LPS, was a second band associated with the degradation of PMCA1/4 present; moreover, the PMCA3 isoform was not identified. An increase in insulin release was observed with the combination of PAO and SA, when compared with PAO. In order to extend this phenomenon associated with the maintenance of [Ca^2+^], no modifications were found in the SERCA2 expression. Remarkably, the cellular internalization of SA was diminished compared to PA (Figure 8D–I).

## 4. Discussion

Considering that the ER lumen is the main site where protein folding and post-translational modifications take place, a decrease of insulin secretion attributed to fatty acid and LPS lipotoxicity could be identified by ER stress through UPR activation. Particularly, XBP1s is associated with the regulation of chaperones and protein degradation [44], relevant to mediate the ER stress that may be related to insulin processing. A similar pattern was observed in the activation of c-Jun, CHOP, and ATF6α transcription factors, which allow reduction of ER stress. Although in our conditions cell viability remains basal even in high concentrations of LPS, this could be associated with the fact that cells are trying to recover homeostasis by activating the UPR.

In an important manner, when main SFAs were evaluated, the most lipotoxic was PA (16C) which, according to our results, is mainly located in the endoplasmic reticulum. Although SA (18C), MA (14C) and LA (12C) were evaluated under the same conditions, possibly the length of PA is the optimal for recognition, and then for cellular internalization. In an attempt to extend the characterization of SFAs on key processes of β-cell physiology, our group has designed new materials based on surfaces functionalized with a layer of a fatty acid covalently bound. Results suggest that mainly PA affects the cell adhesion on surfaces compared to other saturated fatty acids (personal communication).

An association between ER stress and the development of DM2 has been previously described [45]. Nevertheless, no influence of SFAs and LPS on β-cell physiology has been reported hitherto. Our data suggest that LPS activated the UPR pathway, but did not reduce cell viability. However, cell viability was reduced significantly with PA and LPS stimuli, associated with cell internalization of the BDP-PA probe, activating the UPR pathway through CHOP, c-Jun, ATF6α and XBP1s. Considering that unsaturated fatty acids could prevent or even restore cell damage caused by lipotoxicity [46,47], our data suggest that oleic acid could have a partial protective role against lipotoxicity caused by PA/LPS. It is likely that a competition between oleic and palmitic acid for receptors such as FFAR1 or CD36 might occur; in fact, CD36 facilitates fatty acid transport and overexpression affects insulin secretion [48]. In an important way, we previously reported that small molecules with terpene structure could regulate ER stress induced by PA, through inhibition of the eukaryotic initiation factor-4A, with an effect in CHOP and XBP1s expression [49]. Therefore, protein translation is a target that could modulate ER stress induced by lipotoxicity.

Our results showed the activation of the UPR pathway by PA and its accumulation in organelle membranes, mainly in the ER area (Figure 2 and Figure 3), generating stress conditions. Also, we observed a decrease in the PMCA1/4 expression, in correlation with an alteration of insulin secretion. It should be noted that the monoclonal antibody employed here recognizes isoforms 1 and 4, which show a ubiquitous expression. Although PMCA3 isoform has been detected specifically in β-cells by other authors [42], this is a controversial issue since we and others have not detected this isoform. In this work, PMCA3 was not detected at the protein level and, importantly, its absence was confirmed also when transcripts were measured by qPCR in all experimental conditions (data not shown). Regarding other PMCA transcripts expression, the observed pattern corresponds to the maximal expression of *ATP2B1* which is the most abundant in all tissues, but also when high intracellular [Ca^2+^] levels are present. By itself, this finding can be related to the specific PMCA1 affinity for Ca^2+^ that is lower with respect to isoforms PMCA2 and PMCA3; when [Ca^2+^] is elevated in the cytoplasm, cells do not require the function of the sensitive isoforms to extrude calcium, as we have observed before [14]. Since PMCA is responsible for intracellular Ca^2+^ removal and Ca^2+^ entry is a trigger factor for insulin release, a decrease in PMCA expression would be expected to generate the opposite effect. This may be due to other mechanisms responsible for [Ca^2+^] regulation in the β-cells, such as the NCX1 exchanger and SERCA. The possibility of studying the expression of transcripts and activity of these proteins in the near future is open.

In our conditions, SERCA2 shows a basal expression, suggesting the importance of maintaining [Ca^2+^] in the ER. Results suggest that there is not severe alteration in the ER. Importantly, the decrease in insulin release upon treatment with the PA/LPS was accompanied by the decrease in PMCA1/4 expression, wherein the detection of a lower molecular weight band of PMCA was a major finding. This effect could be correlated with the degradation of PMCA due to a proteolytic cleavage, a condition mediated by calpains and caspases under oxidative conditions [40], as corroborated by the formation of RNS. In this sense, a phenomenon related to oxidative phenomenon in β-cells is the lipid peroxidation by the generation of α, β-unsaturated 4-hydroxyalkenals (4-HNE, 4-HHE and 4-DDE) [50]. However, oleic and palmitoleic acid avoided this phenomenon, which could improve under the co-treatment with chalcone and flavone analogs recently synthetized by our group [51]. Interestingly, when the combination of palmitic acid plus LPS and the treatment with palmitoleic acid were tested, the UPR activation was minimal. Specifically, palmitoleic acid promoted an increase in insulin secretion without inducing CHOP over-activation, indicating a possible restorative role.

On the other hand, unlike palmitic acid, stearic acid alone did not cause severe ER stress and cell viability remained in basal conditions. In fact, treatment with SA and PAO increases cell viability; this was corroborated by the null modification in PMCA1/4 expression and insulin release. Therefore, we demonstrated that fatty acids exert a differential effect on the physiology of β-cells, with the length of the hydrocarbon chain a contributing factor. Palmitic acid caused the most harmful condition, affecting cell viability and activating the UPR pathway, wherein the saturation and, specifically, the 16C length chain are critical properties. Also, in combination with LPS, it generated a greater deleterious effect in all evaluated parameters: it decreased insulin release, promoted the proteolytic degradation of PMCA, and increased RNS. By contrast, SA did not cause any significant damage, but it decreased insulin secretion.

However, the effect of oleic acid and palmitoleic acid was different in insulin release, PMCA expression, and Ca^2+^ storage. Oleic acid had the ability to restore insulin release, but the expression of PMCA and NCX1 increased. Nevertheless, PMCA and NCX1 expression decreased with PAO/PA and PAO/LPS treatments, and insulin secretion increased maintaining the basal levels of Ca^2+^. Therefore, oleic acid seemed to play a restorative role for the damage induced by SFAs, mainly ER stress, while palmitoleic acid improves insulin release and has more relevant effects upon intracellular calcium regulatory pumps.

Although free fatty acid proportions in the diet are a condition associated with the development of several diseases associated with alterations in metabolism [16,38,49], it is important to consider the adipose tissue as a buffering of lipid fluxes, which could be impaired in obesity by defects in response to the dynamic metabolism that occurs after meals [52], then modifying the relationship in concentrations of saturated/unsaturated plasmatic fatty acids. Therefore, the characterization of the main effects fatty acids have upon pancreatic β-cell physiology and the UPR pathway, and the repercussion on calcium homeostasis and insulin release, allow us to broaden our understanding of the different roles of fatty acids inside β-cells, which are important metabolites to take into account in explaining the damage and treatment of novel therapeutic targets in T2DM.

## Supplementary Materials

The following are available online at https://www.mdpi.com/2073-4409/8/8/884/s1, Figure S1: LPS treatment increases the signal of DBP-PA on endoplasmic reticulum; Figure S2: Activation of UPR arms induced by PA treatment (300 µM); Figure S3: Full Western-blot of XBP1s, c-Jun, and ATF6α under LPS increasing concentrations (0.1-1000 ng/mL); Figure S4: Full Western blot of PMCA1/4 under different conditions of treatment with stearic acid, oleic acid and LPS.
