# Supplementary material for: Fatty Acid and Lipopolysaccharide Effect on Beta Cells Proteostasis and its Impact on Insulin Secretion
Source: Cells. 2019 Aug 13;8(8):884. doi: 10.3390/cells8080884 (PMC6721695; doi:10.3390/cells8080884)

Supplementary figures

Sup. Fig. 1. LPS treatment increases the signal of DBP-PA on endoplasmic reticulum. Confocal images of β-cells treated with BDP-PA (**A**), ER-tracker probe (**B**) and merge (**C**). Under the same conditions, β-cells treated with BDP-PA plus LPS (**D**), ER-tracker plus LPS (**E**), and the merge (**F**). Bars correspond to 20 μm.

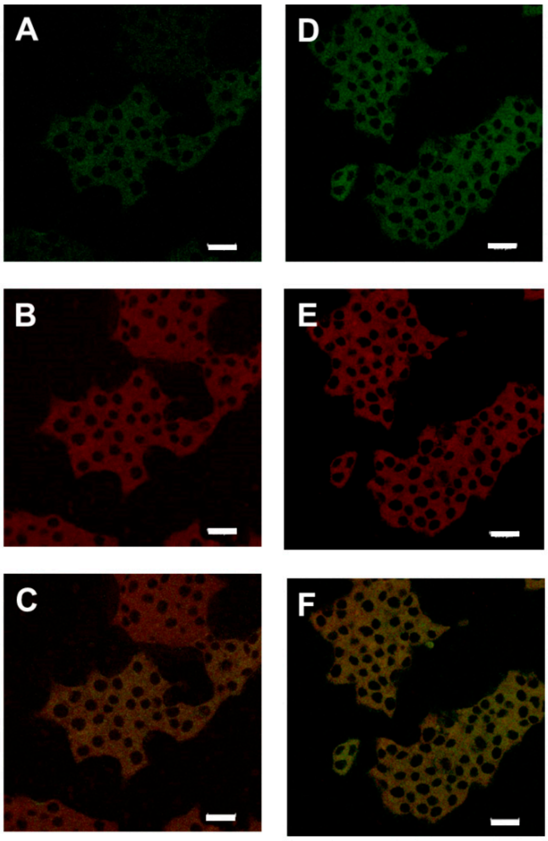

Sup. Fig. 2. Activation of UPR arms induced by PA treatment (300 μM). Tunicamycin (2 μg/mL) was employed as a control of UPR activation. β-actin was used as a loading control.

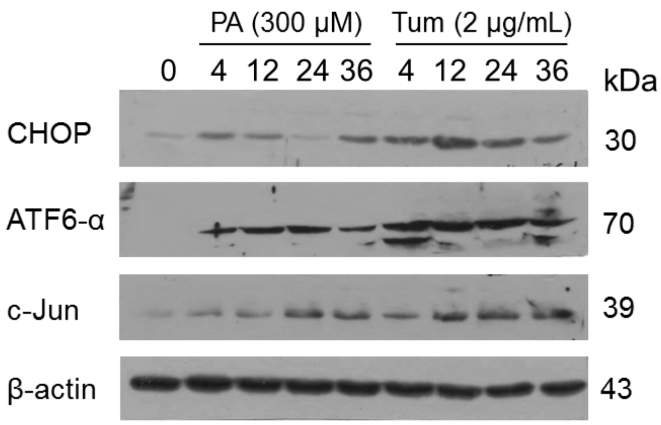

Sup. Fig. 3. Full western-blot of XBP1s, c-Jun, and ATF6α under LPS increasing concentrations (0.1 1000 ng/mL). β-actin was used as a loading control.

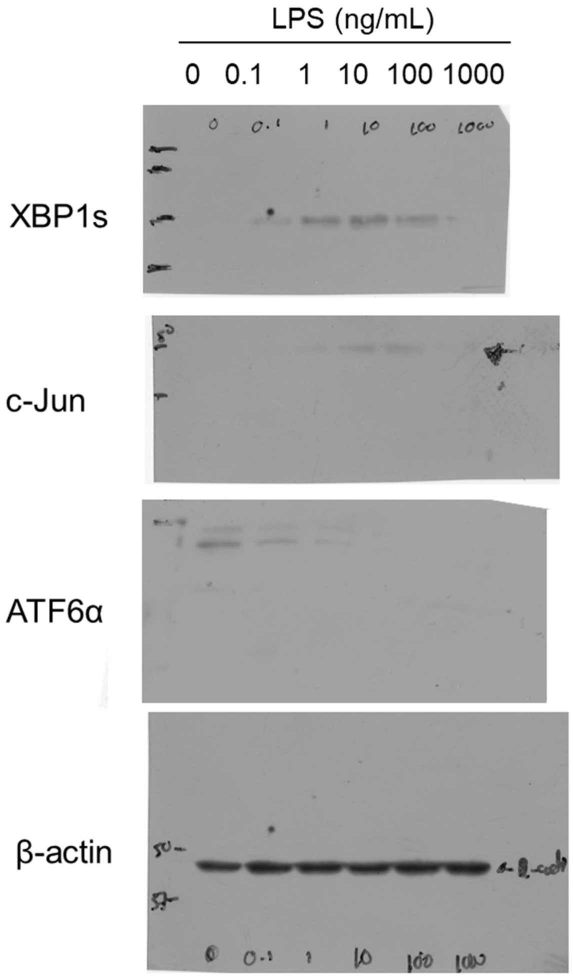

Sup. Fig. 4. Full western blot of PMCA1/4 under different conditions of treatment with stearic acid, oleic acid and LPS.

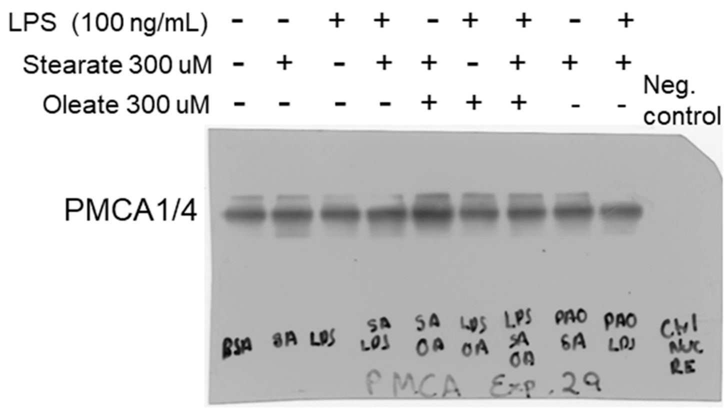

Supplement: Supplementary file 1 [file cells-08-00884-s001.pdf]
